# Optimization of GPC3-specific chimeric antigen receptor structure and its effect on killing hepatocellular carcinoma cells

**DOI:** 10.1080/21655979.2021.1950261

**Published:** 2021-07-14

**Authors:** Jianfeng Zhao, Lijuan Lin, Yihua Luo, Qinghe Cai, Xiaojie Jiang, Changxi Liao, Huimin Wei

**Affiliations:** a Department of Hepatobiliary Surgery, Affiliated Hospital of Putian University, Putian, China; b Department of Rehabilitation, Affiliated Hospital of Putian University, Putian, China; c ImmunoCares Co. Ltd., Guangzhou, China; d The Advanced Institute of Translational Medicine, Tongji University, Shanghai, China

**Keywords:** Glypican-3, chimeric antigen receptor, immunotherapy, hinge region, transmembrane domain

## Abstract

To investigate the effect of optimized GPC3-specific chimeric antigen receptor (GPC3-CAR) structure on killing hepatocellular carcinoma (HCC) cells. We constructed three lentiviral expression vectors with different CAR structures by genetic engineering and molecular cloning techniques. These three CAR structures shared the same intracellular signaling region consisting of 4–1BB and CD3ζ, but had different hinge and transmembrane regions. Specifically, GPC3-O4-CAR contained an optimized CD8α hinge region and a 4–1BB transmembrane domain; GPC3-CD8-CAR contained an optimized CD8α hinge region and a CD8α transmembrane domain; and GPC3-ori-CAR contained an original CD8α hinge region and a 4–1BB transmembrane domain. With similar transfection efficiency, it was observed by fluorescence microscopy that GPC3-O4-CAR expression on the surface of 293 T cells was much higher than those of the other two. Cytotoxicity experiments showed that T or NK cells with GPC3-O4-CAR structure were more lethal and could secrete more IFN-γ than the other two. In conclusion, GPC3-O4-CAR can be efficiently and stably expressed on the cell surface. Moreover, both the killing effect of transduced T and NK cells on GPC3-positive HCC cells and release of IFN-γ are increased.

## Graphical Abstract

**Figure:**
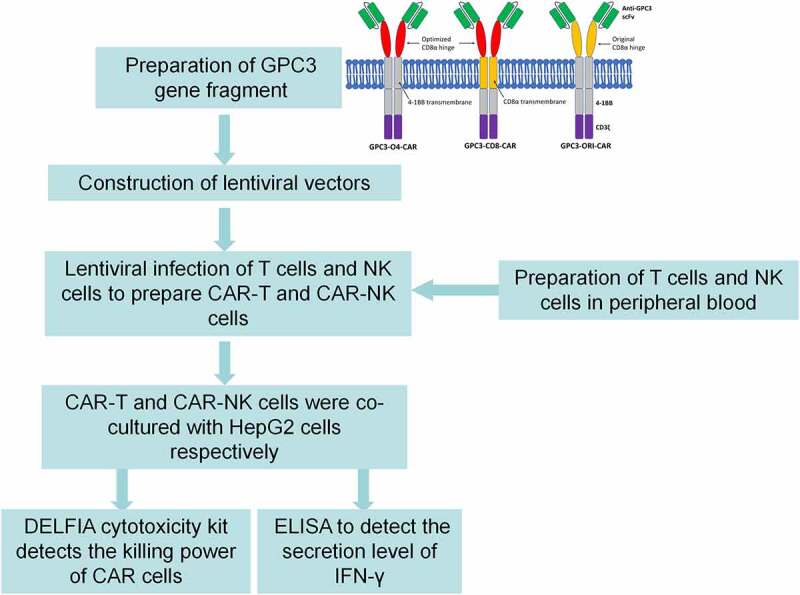

## Introduction

Hepatocellular carcinoma (HCC) is the fourth most common cause of cancer-related death worldwide and currently has the highest incidence in Asia and Africa [1]. In the majority of cases, HCC is diagnosed at an advanced stage, resulting in a poor prognosis [2]. Sorafenib is the first FDA-approved systemic therapy for HCC, extending overall patient survival by approximately 3 months [3]. Recently, lenvatinib, cabozantinib, and ramucirumab have also been approved for patients with HCC who progress after sorafenib treatment [4–6]. Despite the advances in available therapies, effective systemic treatment options for HCC remain limited. Chimeric antigen receptor (CAR)-T cell therapy have received attention from scientists in recent years [7]. CAR-T cell therapy is the modification of T cells, so that T cells gain more precise tumor recognition guidance function, while activating T cells to kill tumor cells more efficiently [8]. In recent years, CAR-T cell therapies have received increasing attention because of their high specificity, tumoricidal efficiency, and sustained efficacy [9,10]. How to obtain CAR cells with high tumoricidal efficiency that can accurately target tumors and less affected by the environmental factors has become a hot spot for cancer therapy.

Effective activation of CAR cells depends on the recognition of tumor-associated antigens (TAA). One of the potential TAA tumor-associated antigens is Glypican-3 (GPC3), belonging to the heparan sulfate proteoglycan family, is a polyglycoprotein anchored to the cell membrane. Studies have revealed that GPC3 is not expressed in normal tissues while is highly expressed in many cancer cells, especially showing a close relation with hepatocellular carcinoma (HCC) [11]. Specifically, GPC3 is highly expressed in the fetal liver, but not in the normal adult liver tissue and other tissues such as heart, brain, and spleen [12,13]. However, its expression recovers to a high level in HCC patients. GPC3 is a specific target for cancer therapy; it has a high detection rate in the early stage of HCC, and with the development of HCC, its detection rate also increases [14]. Gao [15] designed GPC3-targeted CAR-T cells and found that they could effectively eliminate GPC3-positive HCC cells in mice. Maher et al. [16] studied the anti-tumor effect of GPC3-specific CAR-T cells on HCC utilizing patient-derived xenograft models and found that they could significantly inhibit the growth of tumors with high expression of GPC3, further confirming the application prospect of GPC3-specific CAR-T cells in the treatment of HCC. In a clinical experiment of anti-GPC3 monoclonal antibody for HCC patients, Zhu et al. [17] reported that the effectiveness of this treatment was limited by GPC3 expression on the surface of HCC cells, that is, the therapy showed a significant effect when GPC3 was at high levels. Therefore, with GPC3 as a CAR target antibody, CAR that can efficiently and specifically bind to GPC3 can be found. However, the above studies are only for a certain cell such as T cell or NK cell, and two types of cells are not explored at the same time. In this study, GPC3 was used as the target of CAR therapy to optimize the structures of GPC3-specific CAR (GPC3-CAR), so that CAR-GPC3 could be applied to both T cells and NK cells and CAR cells with enhanced therapeutic effects could be obtained. Meanwhile, by optimizing the structures, the expression of GPC3-CAR on the cell surface was increased, thereby improving the affinity and tumoricidal efficiency of CAR cells, and providing a novel option for the treatment of cancer.

## 1 Materials and methods

### 1.1 Reagents

HepG2 and 293 T cell lines were obtained from the Cell Bank of Chinese Academy of Sciences. Top10 Escherichia coli Top10 were stored in our laboratory, and lentiviral vector pLVX-IRES-ZsGreen1 (Cat #. 632,187) was from Clontech. High-fidelity PCR kit (11,304,029), DNA purification kit (K310001), plasmid extraction kit (K210002), FBS (12,676,029), RPMI1640 (C11875500BT), DEME medium (C11995500BT), lipofectamine2000 (11,668,019) were all purchased from Invitrogen. PEG-it (LV810A-1) was purchased from System Biosciences, restriction enzyme (DL5000) and DNA Marker (DL2000) were from TaKaRa, lymphocyte separation medium (LTS1077) was from Tianjin TBD science, CD3 monoclonal antibody (300,314) was from BioLegend, CD3 Microbeads (130–050-101) were from Miltenyi, DELFIA cytotoxicity kit (AD0116) was from PerkinElmer, and IFN-γ ELISA kit (ab46025) was from Abcam. Oligonucleotide primers and rhodamine-labeled C-terminal polypeptides of GPC3 were synthesized by Shanghai Shine Gene Molecular Biotechnology Co., Ltd.

### 1.2 Clinical sample and study design

**Figure 1.**
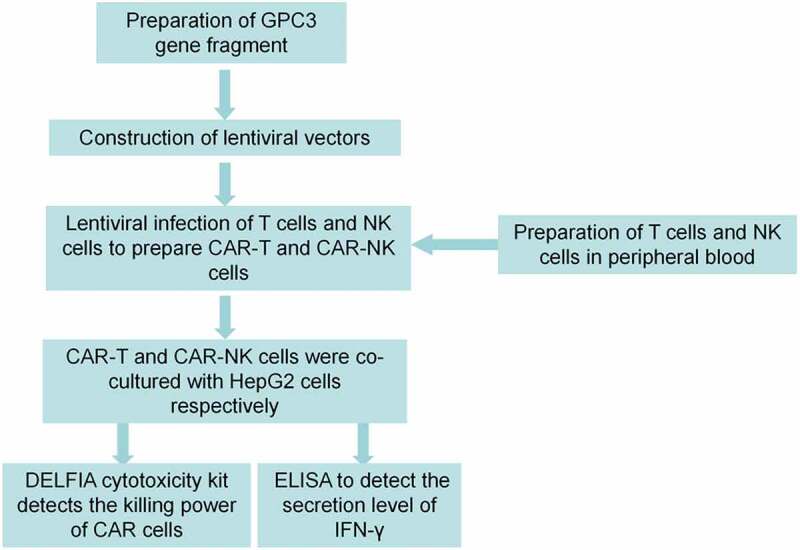
Study design

Peripheral blood mononuclear cells (PBMCs) were derived from healthy volunteers. All participants signed an informed consent form and the study was approved by the Ethics Committee of the Affiliated Hospital of Pu Tian University. Subsequently, the study design is shown in Figure 1.

### 1.3 Preparation of gene fragment and construction of lentiviral vectors

Based on the existing humanized high-affinity GPC3 antigen [16], single chain variable fragment (scFv) was designed and GPC3-O4-CAR, GPC3-CD8-CAR and GPC3-ori-CAR genes were directly synthesized by gene synthesis technique. Then the synthesized CAR genes were inserted into pLVX-IRES-Zsori1 plasmids, respectively, and named as pLVX-GPC3-O4-CAR, pLVX-GPC3-CD8-CAR and pLVX-GPC3-ori-CAR.

### 1.4 Lentivirus production

The 293 T cells with good growth status were digested with trypsin to prepare single cell suspension whose concentration was then adjusted to 4 × 10^5^/mL. Subsequently, 10 mL suspension was taken and placed in a CO_2_ incubator at 37°C for overnight culture. Twenty-four hours later, 3 μg pMD2G, 6 μg psPAX, and 7.5 μg plasmid were added to 150 μLOPTI-MEM and mixed; 25 μL Lipofectamine 2000 was added to 500 μL OPTI-MEM, mixed and then placed at room temperature for 15 min. After that, the plasmid mixture was slowly added to Lipofectamine2000, mixed and placed at room temperature. After 15 min, the plasmid-lipo mixture was added into the petri dish drop by drop and mixed. After 6 hours, the medium was changed to DMEM fresh culture medium containing 10% FBS. On completion of 48 hour-culture, the supernatant containing lentivirus was collected and centrifuged at 1500 g for 10 min to remove cell debris. Then the supernatant was filtered through a membrane (0.45 μm pore size). The filtrate was added with 5 × PEG-it virus concentrate at a ratio of 4:1, placed at 4°C overnight and centrifuged 3200 g for 10 min. On completion of centrifugation, the supernatant was aspirated, and the viral pellet was resuspended with DMEM medium containing 5% FBS. A small amount of virus was taken for titer determination, and the remaining virus was aliquoted and stored at −80°C.

### 1.5 Detection of GPC3-CAR expression on the cell surface

The above three plasmids with different CAR structures were purified and transfected into 293 T cells, respectively. After 24 hours, rhodamine-labeled C-terminal polypeptides of GPC3 (rhodamine+DGMIKVKNQLRFLAELAYDLDVDDAPGNSQATPKDNEISTFHNLGNVHS) were added. Then 1 h later, after washing off the unbound polypeptides with PBS, the cells were observed under a fluorescence microscope.

### 1.6 Preparation of CAR-T and CAR-NK cells

Preparation of CAR-T cells. First, 50 mL of peripheral blood was drawn from volunteers, and then PBMCs were isolated by density gradient centrifugation with lymphocyte separation solution. After being rinsed twice with PBS, T lymphocytes were activated with CD3 monoclonal antibody for 24 hours. The activated T lymphocytes were then resuspended in RPMI 1640 medium containing 100 IU/mL IL-2. Then, lentivirus was added at a ratio of MOI = 5, and medium containing IL-2 was utilized for the following 7-day culture. Finally, CAR-T cells were obtained.

Preparation of CAR-NK cells. Similarly, 50 mL peripheral blood was drawn from the volunteers, followed by isolation of PBMCs by density gradient centrifugation with lymphocyte separation solution. After being rinsed twice with PBS, CD3 Microbeads was utilized to remove T lymphocytes, and then co-cultured with 40 Gy gamma-irradiated B-lymphoblastoid cell lines (LCLs) in RPMI1640 medium containing 200 IU/mL IL-2 for 8 days for activation and proliferation. The proliferated NK cells were resuspended in RPMI 1640 medium containing 200 IU/mL IL-2. Then, lentivirus was added at a ratio of MOI = 5, and medium containing IL-2 was utilized for the following 2-day culture. Finally, CAR-NK cells were obtained.

### 1.7 Detection of tumoricidal efficiency of CAR-T and CAR-NK cells to GPC3-positive hepatocellular carcinoma cells

Tumoricidal efficiency was detected according to the instructions of DELFIA kit. The obtained CAR-T or CAR-NK cells were used as effector cells, and GPC3-positive HepG2 HCC cells as target cells. HepG2 cells were labeled with BATDA for 15 min. After being rinsed with PBS for 3 times, CAR-T or CAR-NK cells and HepG2 cells were co-cultured with effector-to-target ratios of 2.5:1, 5:1, 10:1, 20:1, and 40:1 for 3 hours. The supernatant was then taken and mixed with Eu^2 +^ solution to measure the fluorescence intensity. Finally, the tumoricidal efficiency of CAR-T or CAT-NK cells to HepG2 cells at different effector-to-target ratios was calculated.

### 1.8 Detection of IFN-γ secretion by ELISA

CAR-T or CAR-NK cells with different GPC3-CAR structures were co-cultured with HepG2 cells at a ratio of 1:1, respectively. After 24 hours, the supernatant was taken to detect the IFN-γ concentration by ELISA kit.

### 1.9 Statistical analysis

SPSS 26.0 was adopted for statistical analysis. Measurement data were expressed as mean ± standard deviation (SD), and independent T-test was used for the comparison between two groups. A significant difference was indicated if P < 0.05.

## 2 Results

### 2.1 Construction of lentiviral plasmid

**Figure 2.**
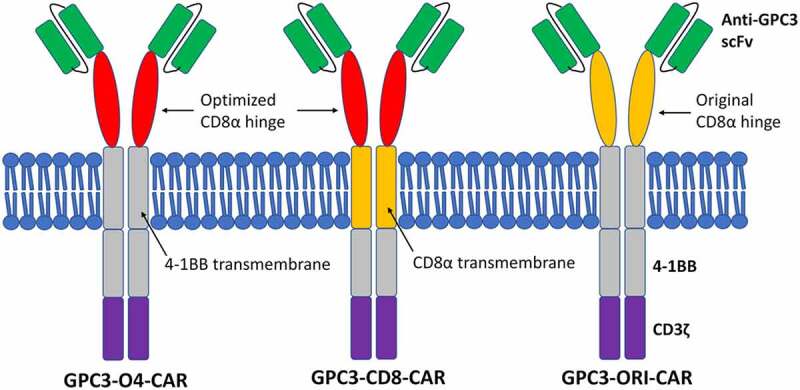
Diagrams of different GPC3-CAR structures

GPC3-CAR was composed of signal peptide, hinge region, transmembrane domain, and intracellular region. The hinge regions of GPC3-O4-CAR and GPC3-CD8-CAR were optimized CD8α hinge regions (sequence: TTTPAPRPPTPAPTIASQPLSLRPEASRPAAGGAVHTRGLDFACD). This meant that the 27th amino acid away from the cell membrane was changed from cysteine to serine, but the ability of C-terminal cysteine near the cell membrane to form dimers did not change. The transmembrane domain of GPC3-O4-CAR was that of 4–1BB (sequence: IISFFLALTSTALLFLLFFLTLRFSVV), while transmembrane domain of GPC3-CD8-CAR was that of CD8α (sequence: IYIWAPLAGTCGVLLLSLVITLYC). The hinge region of GPC3-ori-CAR was original CD8α hinge region and the transmembrane domain was that of 4–1BB (Figure 2).

**Figure 3.**
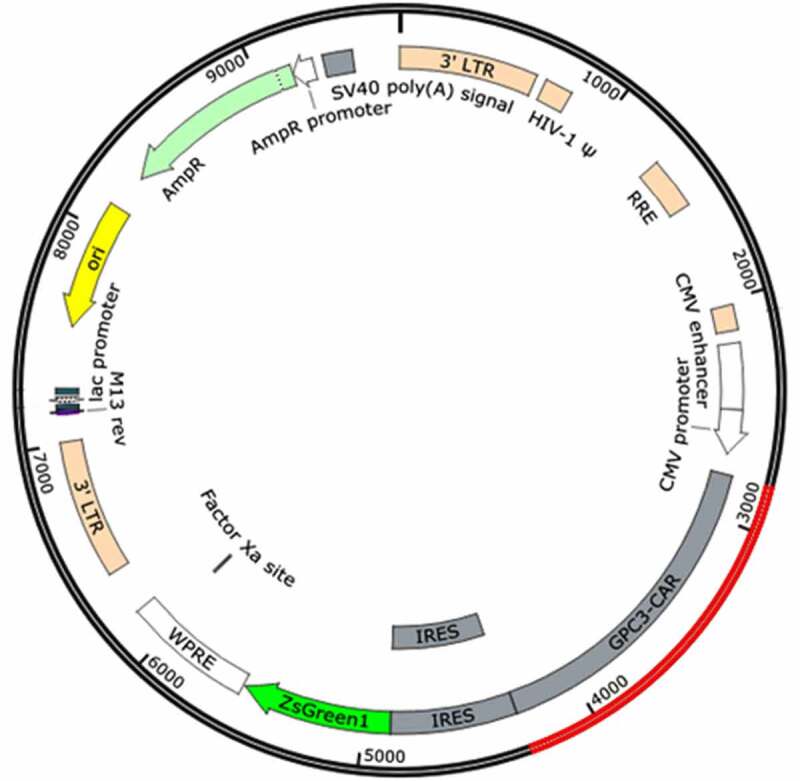
GPC3-CAR expression plasmid for lentiviral preparation

The synthesized CAR genes were inserted into the pLVX-IRES-ZsGreen1 plasmid to obtain three plasmids, pLVX-GPC3-O4-CAR, pLVX-GPC3-CD8-CAR and pLVX-GPC3-ori-CAR. PLVX-IRES-ZsGreen1, a lentivirus-based expression plasmid, belongs to the third generation of self-inactivating lentiviral vector systems. In addition to expressing an inserted foreign gene, PLVX-IRES-ZsGreen1 can simultaneously express green fluorescent protein by IRES to assess transfection efficiency. After synthesis of the CAR gene sequence, it was digested with BamHI and EcoRI and inserted into pLVX-IRES-ZsGreen1 to obtain a plasmid expressing GPC3-CAR (Figure 3).

### 2.2 Effect of different GPC3-CAR structures on GPC3-CAR expression on the cell surface

**Figure 4.**
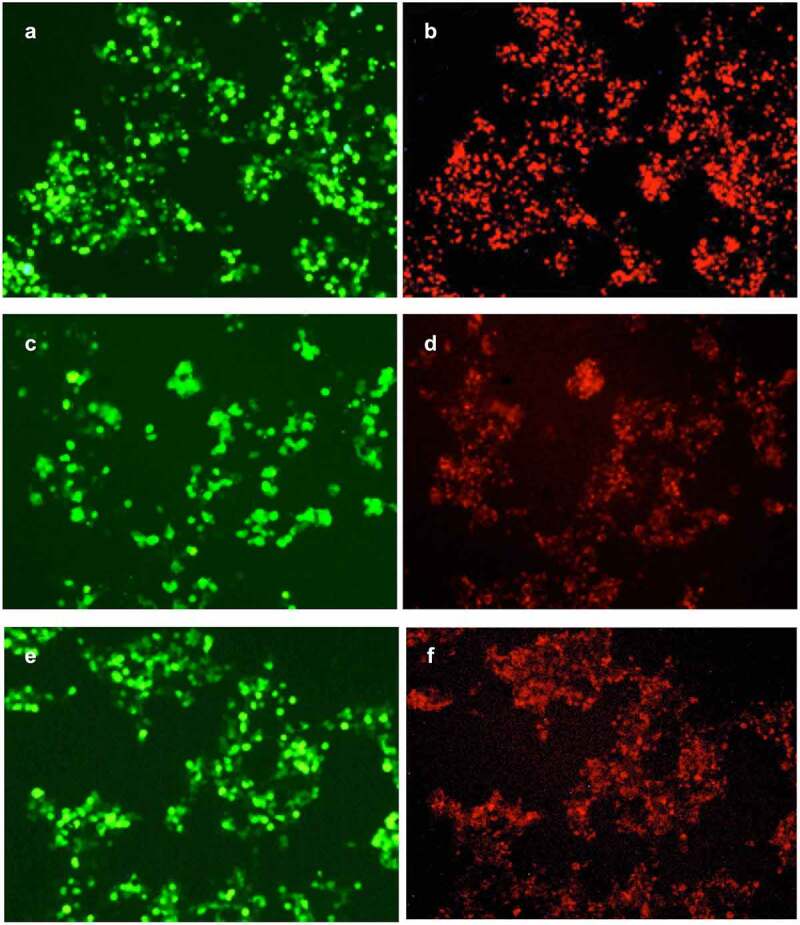
Expression of different GPC3-CAR structures on the cell surface

As shown in Figure 4, the cells transfected with pLVX-GPC3-O4-CAR, pLVX-GPC3-CD8-CAR and pLVX-GPC3-ori-CAR had similar green fluorescence intensity, indicating the similar transfection efficiency. However, the red fluorescence of the cells transfected with pLVX-GPC3-O4-CAR was much stronger than that of pLVX-GPC3-ori-CAR-transfected cells, indicating that GPC3-O4-CAR expression on the cell surface was much higher than that of GPC3-ori-CAR. This results confirmed that the optimized CD8α hinge region in GPC3-O4-CAR was a correct optimization. The red fluorescence of pLVX-GPC3-O4-CAR-transduced cells was also much stronger than that of pLVX-GPC3-CD8-CAR-transfected cells, indicating that GPC3-O4-CAR expression on the cell surface was much higher than that of GPC3-CD8-CAR. This results confirmed that CD8α transmembrane domain was not a correct optimization.

Collectively, optimized CD8α hinge region and 4–1BB transmembrane domain in GPC3-O4-CAR effectively supported the correct folding and expression of GPC3-CAR on the cell surface.

A, C, E: Green fluorescence expression of ZsGreen after transfection with pLVX-GPC3-O4-CAR (A), pLVX-GPC3-CD8-CAR (C) and pLVX-GPC3-ori-CAR (E) plasmids. B, D, F: Measurement of GPC3-CAR expression by rhodamine-labeled polypeptides of GPC3 after transfection with pLVX-GPC3-O4-CAR (B), pLVX-GPC3-CD8-CAR (D) and pLVX-GPC3-ori-CAR (F).

### 2.3 Tumoricidal efficiency of CAR-T and CAR-NK cells

**Figure 5.**
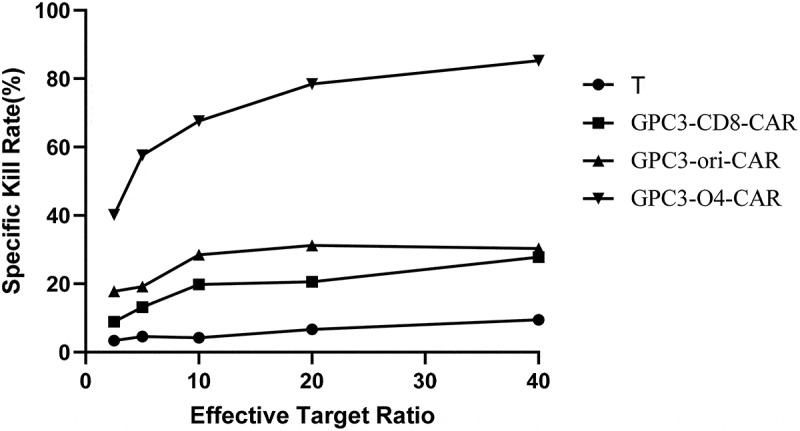
Cytotoxicity activity of T cells expression different GPC3-CAR structures to HepG2 cells

As shown in Figure 5, the kill rate of T cells without transduction to HepG2 cells was less than 10%. However, at effector-to-target ratios of 2.5:1, 5:1, 10:1, 20:1, and 40:1, the kill rates of CAR-T cells with GPC3-CD8-CAR structure were 8.9%, 13.2%, 19.8%, 20.6%, and 27.8%, that of CAR-T cells with GPC3-ori-CAR increased to 17.8%, 19.2%, 28.5%, 31.2%, and 30.3%, and that of CAR-T cells with GPC3-O4-CAR increased to 40.2%, 57.6%, 67.6%, 78.4%, and 85.3%, respectively. Collectively, GPC3-O4-CAR-transfected T cells were more lethal to HepG2 cells than T cells transduced with GPC3-CD8-CAR and GPC3-ori-CAR.

**Figure 6.**
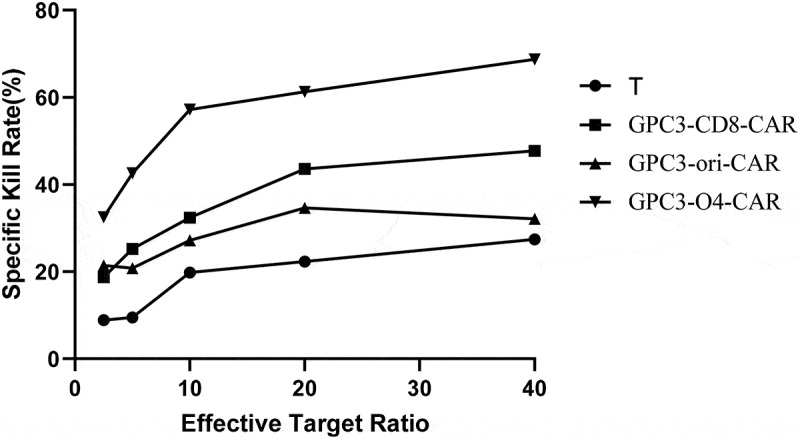
Cytotoxicity activity of NK cells expressing different GPC3-CAR structures to HepG2 cells

As shown in Figure 6, the kill rate of NK cells without transduction to HepG2 cells was less than 30%. However, at effector-to-target ratios of 2.5:1, 5:1, 10:1, 20:1, and 40:1, the kill rates of CAR-NK cells with GPC3-CD8-CAR structure were 18.7%, 25.2%, 32.4%, 43.6%, and 47.7%, that of CAR-NK cells with GPC3-ori-CAR on HepG2 cells was 21.4%, 20.8%, 27.2%, 34.6%, and 32.1%, and that of CAR-NK cells with GPC3-O4-CAR increased to 32.5%, 42.6%, 57.2%, 61.3%, and 68.7%, respectively. Collectively, GPC3-O4-CAR-transfected NK cells were more lethal to HepG2 cells than NK cells transfected with GPC3-CD8-CAR and GPC3-ori-CAR.

### 2.4 Detection of IFN-γ secretion by ELISA

In the cytokine release assay by ELISA, GPC3-CAR-T or GPC3-CAR-NK expressing different GPC3-CAR structures were co-cultured with HepG2 cells at a ratio of 1:1, respectively. After 24 hours, IFN-γ concentration in the supernatant was measured. As shown in Table 1, in both T cells and NK cells, cells with GPC3-O4-CAR structure secreted more IFN-γ than those with GPC3-CD8-CAR and GPC3-ori-CAR, indicating that immune cells expressing GPC3-O4-CAR could be better activated by GPC3 on the surface of cancer cells.

**Table 1.** Secretion of IFN-γ (pg/mL) after stimulating GPC3-CAR-T or GPC3-CAR-NK cells with GPC3

Type of cell	Without transduction	GPC3-O4-CAR	GPC3-CD8-CAR	GPC3-ori-CAR
T cell	1553 ± 126	3876 ± 274*	2497 ± 269^*#^	2655 ± 197^*#^
NK cll	1259 ± 110	4813 ± 378*	3970 ± 362^*#^	3211 ± 353^*#^

Data was presented as mean ± SD. *P < 0.05 *vs*. without transduction group; ^#^P < 0.05 *vs*. GPC3-O4-CAR group.

## 3. Discussion

GPC3 is a polyglycoprotein anchored on the cell membrane. In clinical trials, GPC3-based monoclonal antibody has shown a certain efficacy in HCC treatment, but it still cannot completely eradicate the tumor [18]. So it is a good expansion to use CAR cells to treat HCC with GPC3 as a target. It has been revealed that different CAR-T structures targeting GPC3 have some differences in cytotoxicity to cells and adaptability to the environment [19]. In order to achieve better results with CAR therapy, increasing the number of GPC3-CAR and improving the binding rate of CAR to GPC3 become crucial when GPC3 expression is certain in cancer cells.

CAR is composed of extracellular binding region, hinge region, transmembrane domain and intracellular signaling region [20]. Extracellular region can specifically recognize scFv of tumor-associated antigens, which is non-MHC-restricted [21]. For improving the therapeutic effect of CAR, many studies have designed extracellular binding regions targeting a variety of TAA to upregulate the affinity of antibodies to antigens [22]. Intracellular signaling region can achieve dual activation of costimulatory molecules and intracellular signals, which can allow T cells to continuously proliferate and release cytokines, thereby improving their anti-tumor ability. The optimization of extracellular and intracellular regions enables CAR cells to act more effectively on tumor cells [23]. Hinge region and transmembrane domain connect the extracellular region of CAR with the intracellular region, and anchor the receptor to the T cell membrane. Hinge region and transmembrane domain are essential for the correct folding of CAR molecules and dimer formation, and different single-chain antibodies require different optimization hinge and transmembrane regions to achieve the maximum effects [24]. Appropriate binding region contributes to the effects on tumor cells. Hinge region is an extracellular bridge connecting scFv to the transmembrane region and can spatially assist the extracellular antigen binding domain away from the cell surface for better recognition and binding of related antigens. Transmembrane domain is derived from CD4, CD8α, CD28, 4–1BB, and CD3ζ, which can affect CAR-modified T cells [25,26]. The efficiency of CAR is affected by length of hinge region, interaction interface with the target antigen, target protein density and localization, the selection of costimulatory molecules and the intermembrane distance [27,28]. Therefore, the optimization of CAR structures is the key to CAR treatment.

In this study, we intended to improve the efficiency of CAR by optimizing the hinge and transmembrane regions of CAR. Specifically, first, the 27th amino acid in the hinge region of CD8α away from the cell membrane was changed from cysteine to serine by genetic mutation, and CAR cells with higher killing efficiency were obtained. This may be achieved by the mechanism by which cysteine can form disulfide bonds. Disulfide bonds are crucial for the correct folding, and formation and maintenance of higher order structures of proteins; loss of disulfide bonds or disulfide bond re-formation can regulate proteins to perform specific functions [26]. The change of the 27th amino acid cysteine to serine may avoid the protein folding errors caused by the thiol group of this cysteine and scFv disulfide bonds. This change makes the length of the hinge region more reasonable and improves the efficiency of CAR. Second, we also utilized CD8α transmembrane region to replace the original 4–1BB transmembrane region, but it failed to increase the killing efficiency of CAR, but reduced it a lot. This result indicates that 4–1BB transmembrane region is more appropriate for the whole CAR protein structure and is more conducive to the activity of CAR cells. We finally found that optimized CD8α hinge region and the 4–1BB transmembrane region can increase GPC3 expression on cell surface and the binding rate of CAR to GPC3, and consequently the kill rate of CAR cells to HCC cells.

NK cells, critical immune cells in the human body, have a broad spectrum of anti-tumor effects. NK cells can nonspecifically kill tumor cells, and also have a sound application prospect for adoptive immunotherapy of tumors. Compared with CAR-T, CAR-NK can use allogeneic NK cells, which has strong cytotoxic ability and is easy to prepare on a large scale. In recent years, there are studies on NK-92 cell expressing different CAR for leukemia and solid tumors [29,30]. In this study, different structures of GPC3-CAR were used to co-culture with HCC cells after lentiviral infection of T-cell nuclear NK cells. The results revealed that GPC3-CAR had a strong toxic effect on HCC cells, while increasing the secretion of IFN-γ. Among them, GPC3-O4-CAR bodies of T or NK cells showed the strong cytotoxicity. With this method, the process of collecting PMBCs to prepare T is simplified and the original NK cells as impurities can also be transfected into active ingredients. Thus, the loss during isolation is reduced and the efficiency of peripheral blood use is improved. Collectively, by this method, CAR therapy becomes more economical and effective, and benefits a greater percentage of patients.

This study has some limitations. The CAR was only validated against HepG2 cells, which is somewhat one-sided, and further experiments are needed to verify its efficacy for HCC treatment. In future studies, we need to further validate the killing effect of this CAR on other different HCC cells, and verify the affinity of this CAR with solid tumors, anti-tumor efficacy, survival time in tumor-bearing animals and adverse effects caused in animal experiments one by one, as well as to select suitable drugs that can be used with this CAR for treatment.

## 4. Conclusion

In summary, GPC3-O4-CAR containing the optimized CD8α hinge region and 4–1BB transmembrane region can be efficiently and stably expressed on the cell surface. Moreover, both the killing effect of transduced T lymphocytes and NK cells on GPC3-positive HCC cells and release of cytokines such as IFN-γ are increased. These findings provide more effective data support for clinical application.

## Highlights

1. Three CAR structures (GPC3-O4-CAR, GPC3-CD8-CAR, GPC3-ori-CAR) were constructed.
2. GPC3-O4-CAR can be efficiently and stably expressed on the cell surface.
3. T or NK cells with GPC3-O4-CAR structure were more lethal and could secrete more IFN-γ.
